# Gut Microbiome Homeostasis and the CD4 T- Follicular Helper Cell IgA Axis in Human Immunodeficiency Virus Infection

**DOI:** 10.3389/fimmu.2021.657679

**Published:** 2021-03-19

**Authors:** Olusegun O. Onabajo, Joseph J. Mattapallil

**Affiliations:** ^1^ Laboratory of Translational Genomics, Division of Cancer Epidemiology and Genetics, National Cancer Institute, National Institutes of Health (NIH), Bethesda, MD, United States; ^2^ F. E. Hebert School of Medicine, Uniformed Services University, Bethesda, MD, United States

**Keywords:** HIV, SIV, microbiome, Tfh, IgA, mucosa, GALT, microbial translocation

## Abstract

Human Immunodeficiency Virus (HIV) and Simian Immunodeficiency Virus (SIV) are associated with severe perturbations in the gut mucosal environment characterized by massive viral replication and depletion of CD4 T cells leading to dysbiosis, breakdown of the epithelial barrier, microbial translocation, immune activation and disease progression. Multiple mechanisms play a role in maintaining homeostasis in the gut mucosa and protecting the integrity of the epithelial barrier. Among these are the secretory IgA (sIgA) that are produced daily in vast quantities throughout the mucosa and play a pivotal role in preventing commensal microbes from breaching the epithelial barrier. These microbe specific, high affinity IgA are produced by IgA+ plasma cells that are present within the Peyer’s Patches, mesenteric lymph nodes and the isolated lymphoid follicles that are prevalent in the lamina propria of the gastrointestinal tract (GIT). Differentiation, maturation and class switching to IgA producing plasma cells requires help from T follicular helper (Tfh) cells that are present within these lymphoid tissues. HIV replication and CD4 T cell depletion is accompanied by severe dysregulation of Tfh cell responses that compromises the generation of mucosal IgA that in turn alters barrier integrity leading to commensal bacteria readily breaching the epithelial barrier and causing mucosal pathology. Here we review the effect of HIV infection on Tfh cells and mucosal IgA responses in the GIT and the consequences these have for gut dysbiosis and mucosal immunopathogenesis.

## Introduction

Human and Simian Immunodeficiency Virus (HIV, SIV) infections are associated with dramatic changes in mucosal tissues such as the gastrointestinal tract (GIT) with massive replication and depletion of CD4 T cells during the acute stages of infection (1–17). Viral replication and associated inflammation persists in these tissues over the course of infection that is accompanied by damage to intestinal epithelial barrier, translocation of microbial products, immune activation and progressive loss of CD4 T cells (18–22). Though peripheral tissues experience significant levels of restoration after antiretroviral therapy, viral replication in mucosal tissues continues to persist with incomplete restoration of the immune system to a homeostatic state (23–38). As such, the inflammatory microenvironment in the mucosa remains in a state of high activation that is driven by microbial antigens that traverse the leaky epithelial barrier damaged during infection.

The GIT hosts the largest collection of microbes in the human body with bacteria accounting for a majority of the gut microflora. Studies have estimated that there are over 35,000 bacterial species in the human gut (39), a diversity that is only matched by the sheer magnitude of bacteria colonizing the GIT with estimates ranging from 10^11^ – 10^13^ bacteria (40). Within the GIT, the large intestine accounts for over 70% of the microbes that colonize the human body (41).

The resilience of the gastrointestinal mucosa to withstand continuous exposure to foreign antigens alongside the vast magnitude of microbes and their byproducts is exemplified by the homeostatic state that is maintained throughout life that ensures both intestinal and overall health. This homeostatic balance is maintained by a complex interplay between the host immune system and the gut microbiota that is characterized by a combination of tolerant and antigen specific immune responses that is constantly shaped and modulated by the microbes that reside in the GIT. This symbiotic relationship allows for physiological functions to proceed normally in a tightly controlled immunoregulatory environment. This regulation is essential to prevent any immunopathology that may develop due to responses to continuous exposure to both dietary and microbial antigens.

How the mucosal immune system regulates the mucosal microenvironment and at the same time keeps microbes from crossing the intestinal barrier has been an area of extensive research. In addition to innate mechanisms such as the thick mucus lining the mucosal surface, anti-microbial proteins like defensins, and lectins such as RegIIIγ (42–47), numerous studies have delineated the critical role for antibody responses such as microbe specific secretory IgA (sIgA) in preventing invasive microbes from breaching the epithelial barrier and gaining entry into the submucosa and systemic circulation (48–51).

## IgA and Antimicrobial Immunity

Secretory IgA is the primary immunoglobulin found on mucosal surfaces and exists as a dimer that is bound to each other by the J chain, 15 kD polypeptide (52–55). The J chain binds to cysteine residues in the Fc regions of the heavy chain *via* disulphide bonds (55). Dimeric IgA is transported across the epithelium lining the gut by the polymeric immunoglobulin receptor that binds to IgA on the basolateral side of the epithelium and plays a role in transporting the sIgA across to the apical side and into the gut lumen (56–58). sIgA has significant antimicrobial activity and binds and neutralizes microbes, enhances agglutination of bacteria, and blocks cell adhesion thereby preventing access to the mucosal epithelium (56, 57).

## Mucosal IgA and T- Follicular Helper Cells

Secretory IgA is the most abundant antibody (~ 50 mg/Kg/day) found in the lumen of the GIT with the gut associated lymphoid tissue (GALT) hosting ~ 80% of IgA producing plasma cells in the body (59, 60). Microbe specific sIgA are induced in the Peyer’s Patches and the lymphoid follicles in the GALT following antigen presentation by mucosal dendritic cells (DC) that sample and present microbial antigens to T and B cells. Following differentiation and class switching sIgA is transcytosed across the gut epithelium into the lumen of the GIT (61). sIgA are primarily generated in the Peyer’s patches, isolated lymphoid follicles, and mesenteric lymph nodes that drain the GIT; mice lacking Peyer’s patches have little or no IgA+ plasma cells and sIgA in the gut (62, 63). Isolated lymphoid follicles that develop in the lamina propria are significantly influenced by the microbiota colonizing the gut (64); in the absence of microbiota, germ free mice harbor low numbers of IgA producing plasma cells that secrete IgA that have low affinity to antigens and had failed to undergo somatic hypermutation. In contrast, in the presence of gut microbiota, B cells undergo class switch recombination and somatic hypermutation to produce antigen specific high affinity IgA (65, 66).

sIgA is abundant in mucosa associated lymphoid tissues (MALT) found in other mucosal sites such as the female genital tract (67), the LN that drain the upper and lower respiratory tracts (68). Unlike other MALT, however, Peyer’s patches are unique and specialized for production of isotype switched IgA, while other MALT produce a mix of both IgA and IgG (68–70).

Unlike the isolated lymphoid follicles in the lamina propria that can generate IgA responses in a T cell independent manner, most of the T cell dependent induction of IgA responses occurs within the Peyer’s patches and mesenteric lymph nodes (71–73). T cell dependent induction and differentiation of B cell responses relies on CD4 T cell help (74–78). Though different subsets of CD4 T cells such as Treg (79) and Th-17 (80, 81) have been shown to play a role in the generation of IgA responses in the GIT, T - follicular helper (Tfh) cells are essential for the generation of high affinity IgA responses (82).

Tfh cells are a subset of CD4 T cells that mainly reside in the germinal centers of lymph nodes and provide help to differentiating B cells during the germinal center reaction (83). Tfh cells express CXCR5, PD-1 and ICOS along with BCL6 (83–87), and are a primary source of IL-21, a key cytokine that drives the induction and differentiation of B cell responses (83, 86, 88, 89); mice lacking the IL-21 receptor was found to have defective antibody responses, class-switching, and germinal center (GC) formation (90). A number of other studies have established the central role of Tfh in the induction of high affinity B cell responses (83, 91).

## T- Follicular Helper Cells and HIV Infection

HIV and SIV infections are characterized by loss of CD4 T cells that compromises the immune system with viral reservoirs persisting throughout the life of an infected subject (5, 92–104). Tfh cells residing within the lymph node GC have since been identified as a major source of the persistent latent and productive HIV reservoir (105) due to the inaccessibility of cytotoxic CD8 T cells into B cell follicles within the GC that creates a privileged environment for HIV replication and infection of Tfh cells, the predominant population of CD4 T cells in the follicles (106, 107). Numerous studies have explored the role of lymph nodes in HIV persistence and pathogenesis (108, 109). Perreau et al. (110) reported that HIV infected nonprogressors with low levels of viremia (<1000 HIV RNA copies) harbored high levels of HIV+ Tfh cells as compared to other CD4 T cells subsets. Similarly, SIV infection was associated with significantly high levels of infection in Tfh cells (111–113).

Other studies have reported that HIV specific Tfh significantly expand during chronic HIV infection (114). Similar findings have been reported in a subset of SIV infected macaques where an accumulation of Tfh cells in the lymph nodes that are latently infected is accompanied by higher frequencies of activated GC B cells and SIV specific antibody responses (112, 115–117). Mylvaganam et al. (118) demonstrated that SIV infection was associated with an increase in the frequency of aberrant Tfh in the lymphoid follicles of the rectal mucosa that correlated with lack of viral control. Loss of IL-21+ CD4 T cells was associated with the depletion of other T cell subsets such as Th17 cells and disease progression (119). Numerous studies have documented the depletion of Th17 cells in the mucosa and correlated them with microbial translocation and immune activation (5, 120, 121). A detailed phenotype of Tfh cells that is unique to the GIT is still under investigation. Tfh cells in other specialized lymphoid organs such as the spleen, however express typical Tfh markers, and were shown to decline early during SIV infection (122).

In contrast to the accumulation of Tfh in a subset of infected subjects, numerous studies have reported that HIV and SIV infection were characterized by a depletion of Tfh cells that was associated with altered B cell responses. Mesenteric lymph node Tfh cells were lost very early during SIV infection that was accompanied by a loss of GC and memory B cells (116, 122, 123). Others have reported that the development of both GC reaction and Tfh within these GCs were significantly impaired during HIV and SIV infections that likely contributed to the dysregulation of B cell responses (124–126). Rabezanahary et al. (127) reported a significant decline in Tfh cells in MLN and spleen of SIV infected animals, whereas McGray et al. (128) described a similar decline in the GIT of SIV infected animals during the acute stages of infection. Interestingly, Velu et al. (129) reported that Tfh that expand in the GC during chronic infection harbor a Th-1 bias that differed from conventional Tfh cells raising the possibility that accumulation or depletion of Tfh cells may depend on factors that drive their differentiation.

Despite early antiretroviral therapy, Tfh cells remain major reservoirs in lymphoid tissues and peripheral blood (127, 130, 131). Studies have shown that Tfh were a major source of replication competent HIV and persistent HIV-1 transcription in treated aviremic patients on long term HAART (132). Yu et al. reported that Tfh were enriched for replication competent X4 tropic HIV virus and correlated with progression of disease (133). HIV specific Tfh levels have been shown to significantly correlate with plasma HIV specific antibody levels during HAART (134). Early initiation of HAART maintained HIV-1-reactive memory B cells and Tfh in the gut (135). Rabezanahary et al. (127) reported that Tfh CD4+ T cells in the mesenteric lymph nodes harbor significant levels of viral DNA and RNA in SIV infected rhesus macaques treated with antiretroviral therapy suggesting that Tfh niches that reside in the lymph node GCs were major sites of viral persistence and like other sites are dysregulated during infection. Thornhill et al. (136) demonstrated that CD32 high doublets in the GALT of HIV infected subjects undergoing antiretroviral therapy were primarily composed of Tfh cells that correlated with high levels of rectal HIV DNA.

## Mucosal B Cell Responses and HIV Infection

Mucosal IgA plays a critical role in protecting the mucosa from invasive bacteria that colonize the GIT (137). IgA are primarily produced in a T- cell dependent manner within the Peyer’s Patches and mesenteric lymph node GC with Tfh driving the differentiation, maturation and class switching on B cells (90, 138–142). Chaoul et al. (143) examined T cell dependent IgA responses in the Peyer’s Patches and isolated lymphoid follicles in SIV infected rhesus macaques and detected no IgA+ plasma cells in the GC at these sites during acute infection that was accompanied by a significant decrease in the amount of IgA detectable in the duodenum and ileum. Rather surprisingly, both GC and Tfh within the Peyer’s Patches and isolated lymphoid follicles were functional and intact. Schäfer et al. (144) reported that the amount of total intestinal IgA decreased and remained low during the first few months after SIV infection. Likewise, numerous studies have reported low levels of IgA in mucosal secretions of HIV infected subjects (145–151) whereas Scamurra et al. (152) showed that low levels of IgA in intestinal secretions during HIV infection was associated with lower frequencies of IgA plasma cells in the GIT. It is highly likely that HIV infection significantly impairs B cell differentiation and IgA class switching in the GALT as reported by Xu et al. (153). Levesque et al. (154) reported that GALT GC display significant levels of apoptosis during HIV infection that do not effectively reconstitute after antiretroviral therapy (155). Given the central role of Tfh cells in this process, the loss or dysregulation of Tfh cells during HIV infection likely contributes to B cell dysfunction in the gut that in turn affects the protective mechanisms at the mucosal epithelial barrier. In line with this argument, Hel et al. (123) demonstrated that HIV infected subjects display lower gut microbiota specific IgA and IgG responses in the GIT that likely contributes to microbial translocation and chronic immune activation. Numerous studies have reported the breakdown of gut epithelial barrier during HIV and SIV infections (25, 121, 156–161).

## Gut Microbiota and T - Follicular Helper Cells

The decrease in intestinal IgA is consequential given its central importance in preventing gut microbes from accessing the intestinal epithelium. Gut commensal bacteria and their metabolites play a role in inducing effector T cells and maintaining immune homeostasis in the GIT (162–167). Hegazy et al. (168) reported that gut microbiota reactive CD4 T cells in intestinal mucosa are readily detectable in healthy humans and were a normal feature of the repertoire of CD4 T cells. Likewise, the development of IgA responses is significantly influenced by the microbiota that colonize the gut (65). Large numbers of Tfh cells reside in the GC of Peyer’s patches where gut microbes and their byproducts continuously shape the GC reaction and production of mucosal IgA (169, 170) that in turn regulates gut microbiome interactions with the host. Maruya et al. (171) reported that PD-1 deficiency alters the phenotype of Tfh cells leading to the induction of IgA with reduced capacity to bind gut bacteria that in turn leads to alterations in the composition of the commensal bacteria in the GIT. Loss of PD-1 was also associated with an increase in dysfunctional Tfh cells in the Peyer’s patches that is characterized by reduced production of IL-21, increased secretion of IFNγ, and production of IgA with poor affinity maturation (172). Low affinity IgA responses were accompanied by a profound loss of “healthy bacteria” such as Bifidobacterium and Bacteroides, and an increase in *Enterobacteriaceae* (172). Others (173) have shown that Tfh cells within the Peyer’s Patches play an essential role in systemic arthritis induced by segmented filamentous bacteria by inhibiting IL-2 signaling with DCs contributing to SFB-mediated Tfh cell induction and IL-2 receptor α (IL-2Rα) suppression. IL-2 signaling pathway has been shown to inhibit Tfh cell differentiation by altering Bcl-6 expression (174, 175). Impairment of Tfh cells, either due to lack of inhibitory co-receptor PD-1 or ATP-gated ionotropic P2RX7 receptors, was found to alter the gut commensal bacteria (172, 176). Micci et al. (119) reported that depletion of IL-21 producing CD4 T cells was associated with progression of SIV disease. Taken together, these findings suggest that compromise of mucosal Tfh cells and high affinity IgA responses probably play a role in altering the composition of gut microbiota during HIV infection.

HIV infection is associated with changes in the composition of the gut microbiota (22, 177, 178) characterized by a decrease in *Bacteroides and Firmicutes* and an increase in the prevalence *Proteobacteria (* 179–181). Others have reported alterations in microbial richness (182) and *Prevotella* during chronic HIV infection (181, 183–186). Gut dysbiosis has been reported in SIV infected rhesus macaques that was partially restored with combination antiretroviral therapy (187). SIV infected macaques were found to display an expansion of enteropathogens during advanced stages of disease (188), and macaques with severe disease displayed changes in their bacterial diversity that was characterized by an altered abundance of *Enterobacteriaceae* and *Moraxellaceae* similar to that of HIV infected patients who have low CD4 T cell counts. Lower microbial richness and diversity was associated with poor CD4 T cell reconstitution in HIV infected subjects undergoing treatment with HAART (189, 190). Numerous studies have suggested that some level of gut dysbiosis persists during HAART. Likewise, Tfh cells dysregulated during HIV infection are not fully restored to their healthy state during HART suggesting that B cell dysfunction is likely to persist during therapy that in turn could lead to inadequate protection of the epithelial barrier.

In conclusion, the dysregulation of Tfh cells that compromises B cell responses especially the secretion of microbe specific IgA is one of the likely drivers of gut microbial dysbiosis during HIV infection. This dysregulation that accompanies high levels of HIV replication, loss of mucosal CD4 T cells, breakdown of the integrity of epithelial barrier significantly alters mucosal immune homeostasis leading to microbial translocation, immune activation and progression of disease. Strategies that can preserve and maintain Tfh responses in the mucosa could potentially restore high affinity mucosal IgA that could aid in protecting the mucosal epithelial barrier from invasive dysbiotic bacteria.

## Author Contributions

OO and JM wrote the manuscript. All authors contributed to the article and approved the submitted version.

## Conflict of Interest

The authors declare that the research was conducted in the absence of any commercial or financial relationships that could be construed as a potential conflict of interest.
